# Data set of *Aspergillus flavus* induced alterations in tear proteome: Understanding the pathogen-induced host response to fungal infection

**DOI:** 10.1016/j.dib.2016.11.003

**Published:** 2016-11-09

**Authors:** Jeyalakshmi Kandhavelu, Naveen Luke Demonte, Venkatesh Prajna Namperumalsamy, Lalitha Prajna, Chitra Thangavel, Jeya Maheshwari Jayapal, Dharmalingam Kuppamuthu

**Affiliations:** a Department of Proteomics, Aravind Medical Research Foundation, Dr. G. Venkataswamy Eye Research Institute, Aravind Eye Care System, Madurai, Tamil Nadu, India; b Cornea Clinic, Aravind Eye Hospital, Aravind Eye Care System, Madurai, Tamil Nadu, India; c Department of Ocular Microbiology, Aravind Eye Hospital, Aravind Eye Care System, Madurai, Tamil Nadu, India

**Keywords:** Mycotic keratitis, Tear, Inflammation, Proteomics, Glycosylation, Complement, NETosis, Wound healing

## Abstract

Fungal keratitis is one of the leading causes of blindness in the tropical countries affecting individuals in their most productive age. The host immune response during this infection is poorly understood. We carried out comparative tear proteome analysis of *Aspergillus flavus* keratitis patients and uninfected controls. Proteome was separated into glycosylated and non-glycosylated fractions using lectin column chromatography before mass spectrometry. The data revealed the major processes activated in the human host in response to fungal infection and reflected in the tear. Extended analysis of this dataset presented here complements the research article entitled “*Aspergillus flavus* induced alterations in tear protein profile reveal pathogen-induced host response to fungal infection [1]” (Jeyalakhsmi Kandhavelu, Naveen Luke Demonte, Venkatesh Prajna Namperumalsamy, Lalitha Prajna, Chitra Thangavel, Jeya Maheshwari Jayapal, Dharmalingam Kuppamuthu, 2016). The mass spectrometry proteomics data have been deposited in the ProteomeXchange Consortium via the PRIDE partner repository with the dataset identifier PRIDE:PXD003825.

**Specifications Table**

**Table:** 

Subject area	Biology
More specific subject area	Proteomics of mycotic keratitis
Type of data	Raw/processed data files from LC-MS/MS
How data was acquired	Using Thermo Easy nLC 1000 (Thermo, USA) coupled to Orbitrap Velos Pro mass spectrometer (Thermo, USA)
Data format	Instrument generated raw.files, Proteome Discoverer v1.4 processed.msf files
Experimental factors	Control and patient tear were collected and concentrated by ultrafiltration using a 3 kDa cut-off filter. Lectin affinity chromatography was used to separate glycoproteins before mass spectrometry.
Experimental features	Protein fractions were resolved on 1D SDS-polyacrylamide gel, and the entire lane was sliced into segments and used for in-gel tryptic digestion. Extracted peptides were purified before mass spectrometry using C18 tips.
Data source location	Aravind Medical Research Foundation, Madurai, India.
Data accessibility	The mass spectrometry proteomics data have been deposited to the ProteomeXchange Consortium via the PRIDE partner repository with the dataset identifier PXD003825

**Value of the Data**

• This dataset on tear proteins will be of value for those who are employing tear proteomics approach to understand the fungal and bacterial infections of the human eye.
• The data shows how fungal infection modulates host immunity, which could be studied using readily available tear.
• This dataset provides an insight into the various antifungal defense mechanisms elaborated in the tear which forms the first line of defense against infections.

## 1 Data

**Fig. 1:**
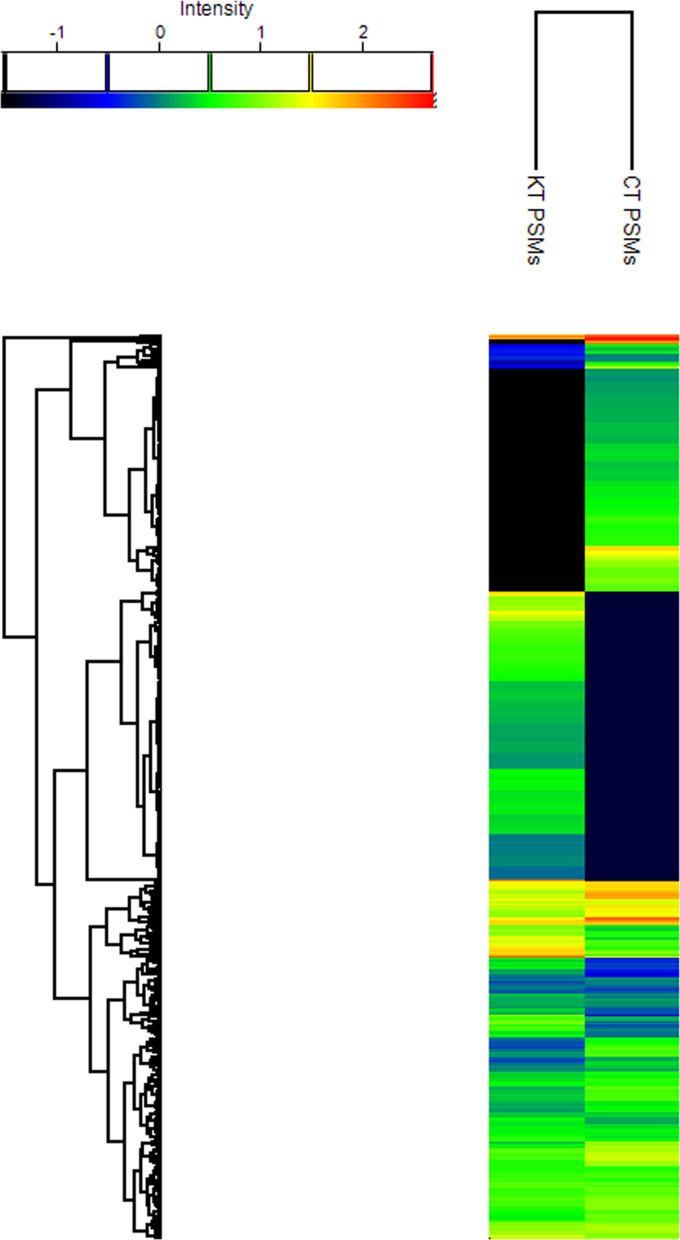
Hierarchical clustering analysis and heatmap based on normalized PSM data across keratitis patient and control sample datasets. KT, keratitis tear; CT, control tear.

**Fig. 2:**
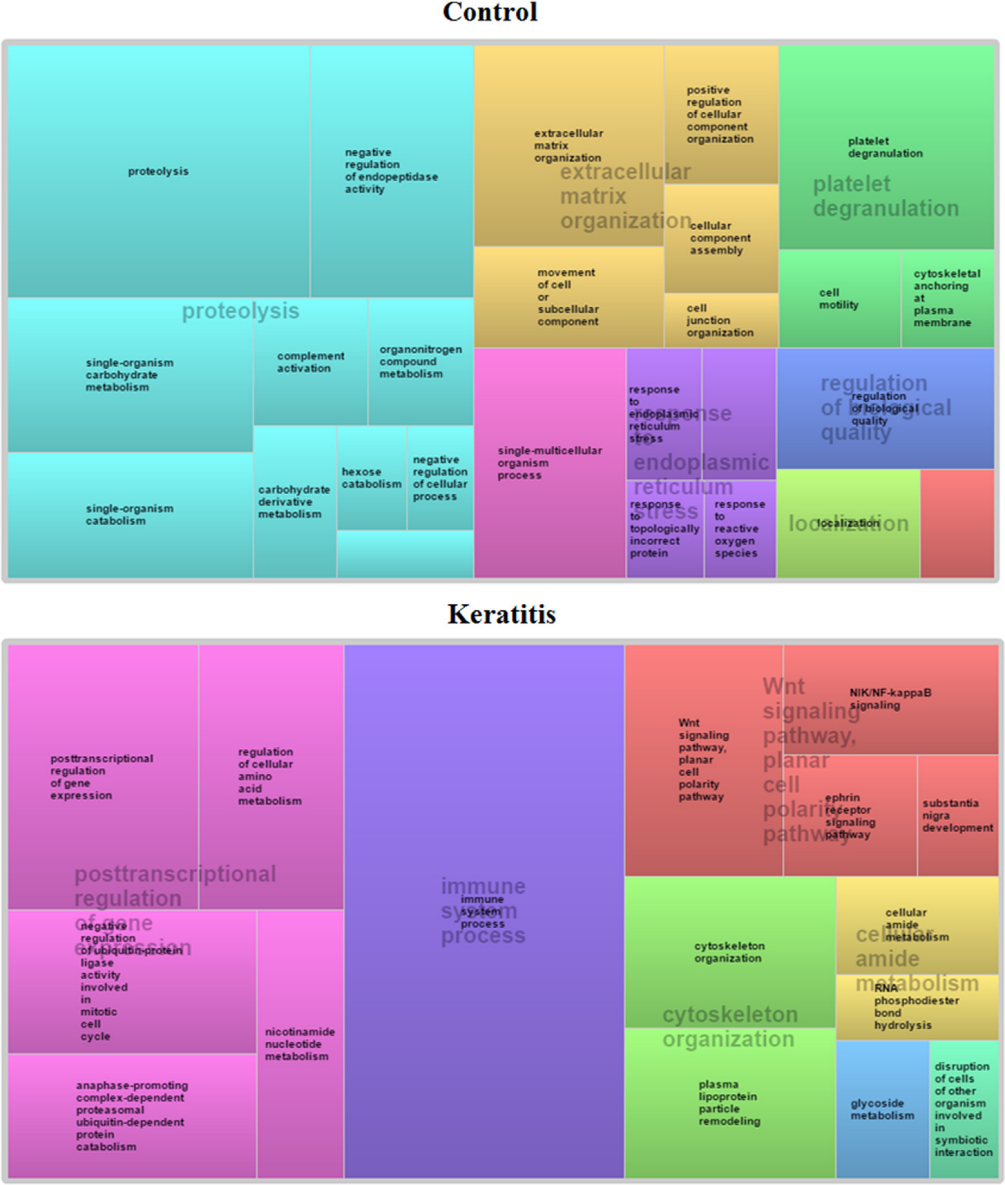
TreeMap view of the enriched GO biological process in control and keratitis datasets. Based on the structure of the GO hierarchy, the terms are clustered by colour and the size of the rectangle is based on the absolute log10 *p*-value.

**Fig. 3:**
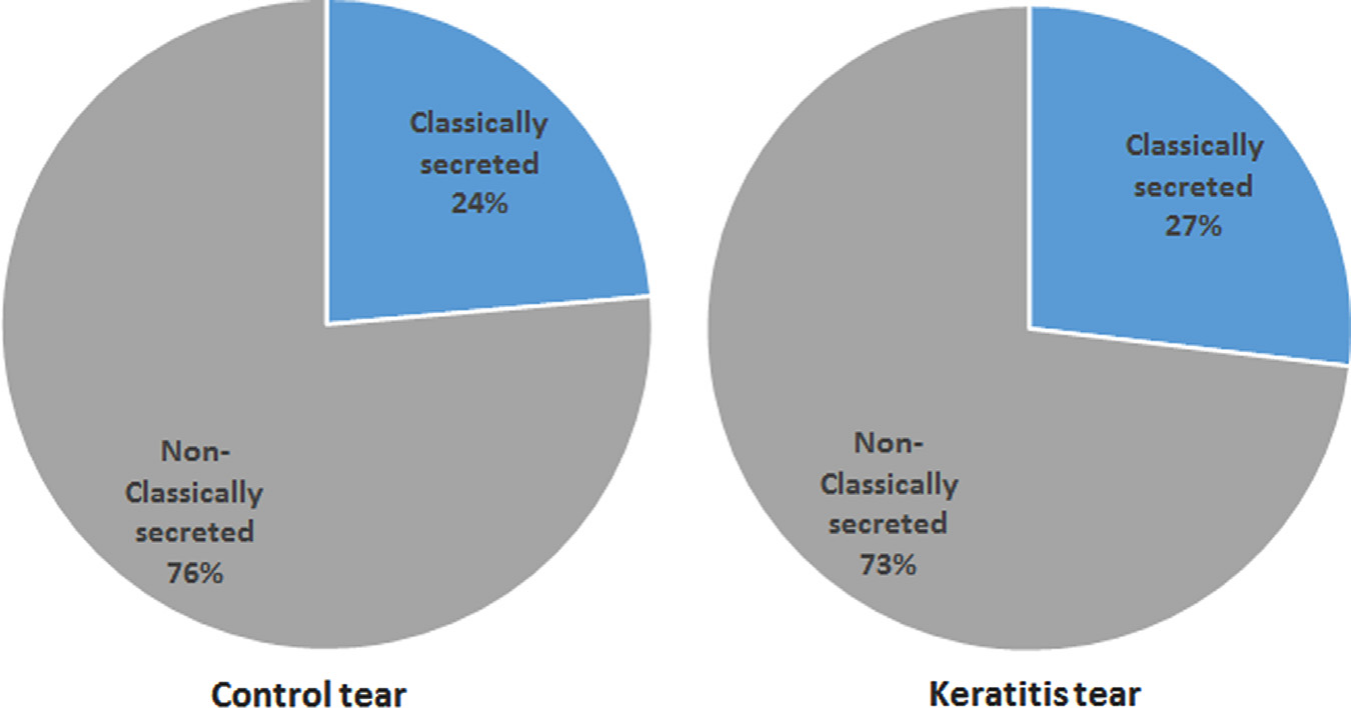
Comparison of the classically and non-classically secreted tear proteins between the control and keratitis tear datasets.

Fig. 1 shows the hierarchical clustering of the datasets comparing the global protein expression across the keratitis patient tear and control tear and is represented as a heat map plot. Protein lists from keratitis and control tear were analyzed for functional significance using g:Profiler[2]. g:Cocoa was used to retrieve significant GO terms, KEGG and REACTOME pathways and compare enrichments between keratitis and control datasets (Supplemental Fig. 1). The significantly enriched GO categories were imported to REVIGO [3] for visualization as shown in Fig. 2. The tear proteins identified were analyzed for the presence of the classical secretory signal using the MetazSecKB secreteome database [4] to determine the percentage of tear proteins that are secreted via the classical pathway. Fig. 3 shows the distribution of tear proteins that are classically and non-classically secreted. The mechanism of secretion of proteins without the signal sequences is not known.

## 2 Experimental design, materials and methods

### 2.1 Tear collection and processing

Reflex tears (50–150 µl) were collected from *Aspergillus flavus* keratitis patients and healthy individuals. The samples were centrifuged briefly to remove cellular debris and snap-frozen in liquid nitrogen until further analysis. All tear samples were collected from patients before the start of anti-fungal treatment. Tear samples were pooled and the protein concentration was estimated using the Bradford׳s method [5]. Tear proteins were prefractionated by 12.5% SDS-PAGE. N-linked glycoproteins in the tear from control and patients were fractionated using ConA lectin spin column as described in Ref. [1]. The flow-through with the non-N-glycosylated proteins and the eluate with the N-glycosylated proteins were desalted and concentrated by ultrafiltration. Protein samples were reduced, alkylated and separated on SDS- PAGE. After visualization of the proteins with coomassie blue, the entire lane was cut separately and sliced into 26 equal sized bands. The proteins in each band were destained and subjected to in-gel tryptic digestion as described earlier [6]. After 16 h of tryptic digestion, the peptides were recovered, dried and desalted using C18 tips (Pierce) as per the manufacture׳s instruction.

### 2.2 LC-MS/MS analysis of tryptic peptides

Tryptic peptides resuspended in 0.1% formic acid and 5% acetonitrile were analyzed by nano-RPLC-MS/MS using an Easy nLC 1000 (Thermo, USA) coupled to Orbitrap Velos Pro mass spectrometer (Thermo, USA). A pre-column (C18, 3 μm particle size, 100 Å, 75 μm × 2 cm) followed by a capillary RSLC column (C18, 2 μm, 100 Å, 75 μm x 50 cm or 15 cm) were used for the separation of peptides using a linear gradient programme (5%–30%. ACN) at a constant flow rate of 200 nL/min.

### 2.3 Data acquisition

Positive mode electrospray ionization with an ion spray voltage of 2.4 kV and a capillary temperature of 200 °C with an RF lens voltage of 69 and maximum injection time of 50 ms. The LTQ was calibrated using the positive ion calibrant solution (Pierce) and tuned to optimize the response of ions at m/z of 524 for the tetrapeptide Met–Arg–Phe–Ala. The acquisition was performed using Nth Order Double Play mode with Xcalibur software (Thermo Scientific, USA). Full scan profile mass spectra were acquired over an m/z of 400–2000 Da at a frequency of 1 spectrum every sec. Top 10 intense ions were targeted for MS/MS under an isolation width of 2 m/z units in CID mode with a collision energy of 35. Switching criteria were set to include ions greater than m/z 400 and smaller than m/z 2000 with the charge state of 2–5 and an abundance threshold of more than 500 counts and the target ions were excluded for 30 s with a repeat duration of 30 s and repeat count set one.

### 2.4 Data processing

**Fig. 4:**
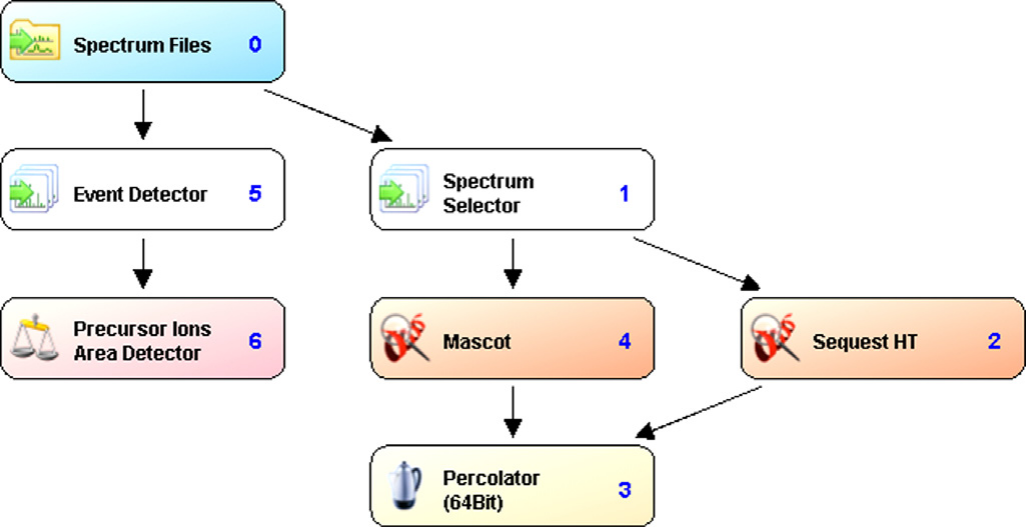
Workflow followed for protein identification using proteome discoverer. Raw files generated by the Orbitrap Velos Pro mass spectrometer were searched against the *Human* protein sequences from UniProt using two different search algorithms. The PSMs from each search result was subsequently validated by the Percolator algorithm.

All MS/MS raw data acquired from Orbitrap Velos Pro mass spectrometer were analyzed by Proteome Discoverer v1.4 following the workflow is shown in Fig. 4. Two search engines, Mascot (Matrix Science, London, UK; version 2.4.1.0) as well as an inbuilt SequestHT algorithm were set up to search a database containing the complete human proteome (including the isoforms) downloaded from the UniProt database on 31th July 2013 (141130 entries) and its Decoy database. The database search was performed with the following parameters: peptide tolerance of 10 ppm and fragment tolerance of 0.60–0.80 Da. The peptide length was restricted to a minimum of six amino acids and a maximum of 144 amino acids for the database search and allowing for two missed cleavages. Cysteine carbamidomethylation was given as fixed modification while methionine oxidation, N-terminal acetylation and phosphorylation (S, T, Y) as variable modifications. The peptide spectrum matches (PSMs) from SequestHT and Mascot were post-processed using the Percolator algorithm [7]. Those peptides having a q-value lesser than the threshold of 0.01 were considered for protein identification. The search result data from all bands from a sample were combined to generate a multi-consensus report to get a non-redundant list of identified proteins. Peptides were filtered for high peptide confidence as well as for rank one. And, proteins identified by one peptide and at least two PSMs were taken as reliable identification for further data interpretation. The mass spectrometry proteomics data have been deposited to the ProteomeXchange Consortium [8] via the PRIDE partner repository with the dataset identifier PXD003825.

### 2.5 Global protein expression profile

After combining the results of the individual replicates in the control and keratitis samples by summing up the PSMs. The missing values were replaced with a constant value of 0.1. The PSM values were log2 transformed after z-score normalization of the data. The normalized PSM values were then taken for hierarchical clustering using Euclidean distances.

### 2.6 Functional enrichment analysis

The significantly enriched GO terms and pathways from KEGG and REACTOME was obtained using the g:Cocoa multi-query algorithm from g:profiler. The “Best per parent (moderate filtering)” option under “Hierarchical filtering” was selected in order to select only the most statistically significant term from a particular hierarchical group. The “order of total significance” option under “Comparison methods” was selected to evaluate the total statistical significance between the control and keratits datasets.

## Transperancy document Supplementary material

.

Supplementary Fig 1. Functional profiling results from g:Cocoa. The colour shows the strength of the statistics.
