# Supplementary material for: Data set of Aspergillus flavus induced alterations in tear proteome: Understanding the pathogen-induced host response to fungal infection
Source: Data Brief. 2016 Nov 9;9:888–94. doi: 10.1016/j.dib.2016.11.003 (PMC5109265; doi:10.1016/j.dib.2016.11.003)
Supplement: Supplementary file 1 — Supplementary material [file mmc1.docx]

**Conflict of Interest Disclosure**

The authors have nothing to disclose.
