# Supplementary material for: Data set of Aspergillus flavus induced alterations in tear proteome: Understanding the pathogen-induced host response to fungal infection
Source: Data Brief. 2016 Nov 9;9:888–94. doi: 10.1016/j.dib.2016.11.003 (PMC5109265; doi:10.1016/j.dib.2016.11.003)
Supplement: Supplementary file 2 — Supplementary Fig 1. Functional profiling results from g:Cocoa. The colour shows the strength of the statistics. [file mmc2.pdf]

source	term name<br>Gene Ontology (Biological process)	term ID	n. of term genes	corrected p-value	KT	CT
BP	pyridine nucleotide metabolic process	GO:0019362	126	6.30e-04	23	
BP	cell redox homeostasis	GO:0045454	65	1.92e-02		14
BP	response to endoplasmic reticulum stress	GO:0034976	256	6.81e-03		33
BP	Wnt signaling pathway, planar cell polarity pathway	GO:0060071	109	7.37e-06	24	
BP	RNA phosphodiester bond hydrolysis	GO:0090501	115	3.21e-02	19	
BP	ephrin receptor signaling pathway	GO:0048013	84	5.42e-03	17	
BP	NIK/NF-kappaB signaling	GO:0038061	106	5.01e-04	21	
BP	regulation of cellular amino acid metabolic process	GO:0006521	54	4.58e-06	17	
BP	killing of cells in other organism involved in symbiotic interaction	GO:0051883	12	4.79e-02	6	
BP	negative regulation of ERBB signaling pathway	GO:1901185	44	4.22e-02		11
BP	anaphase-promoting complex-dependent catabolic process	GO:0031145	77	4.64e-05	19	
BP	substantia nigra development	GO:0021762	44	4.36e-02	11	
BP	negative regulation of ubiquitin-protein ligase activity involved in mit ...	GO:0051436	71	1.08e-05	19	
BP	plasma lipoprotein particle remodeling	GO:0034369	24	3.82e-05	11	
BP	phosphatidylcholine metabolic process	GO:0046470	67	2.95e-02	14	
BP	positive regulation of ubiquitin-protein ligase activity involved in reg ...	GO:0051437	76	3.68e-05	19	
BP	immune system process	GO:0002376	2404	1.58e-21	245	
BP	posttranscriptional regulation of gene expression	GO:0010608	453	8.19e-08	61	
BP	cytoskeleton organization	GO:0007010	1037	3.59e-05	99	89
BP	glycoside metabolic process	GO:0016137	15	1.61e-02	7	
BP	cellular amide metabolic process	GO:0043603	936	6.32e-03	84	
BP	macromolecular complex remodeling	GO:0034367	24	3.82e-05	11	
BP	high-density lipoprotein particle clearance	GO:0034384	9	5.01e-03	6	

source	term name<br>Gene Ontology (Cellular component)	term ID	n. of term genes	corrected p-value	KT	CT
CC	cell surface	GO:0009986	719	4.02e-03		69
CC	cell projection	GO:0042995	1755	3.14e-04	144	136
CC	cytosol	GO:0005829	3316	1.58e-34	342	264
CC	extracellular exosome	GO:0070062	2724	1.29e-196	538	438
CC	proteasome core complex	GO:0005839	22	6.07e-07	12	
CC	secretory granule lumen	GO:0034774	85	1.67e-07	23	23
CC	myelin sheath	GO:0043209	171	2.44e-05	30	25

source	term name<br>Gene Ontology (Molecular function)	term ID	n. of term genes	corrected p-value	KT	CT
MF	GDP binding	GO:0019003	52	4.35e-02	12	
MF	arylesterase activity	GO:0004064	6	7.38e-03	5	
MF	fatty acid binding	GO:0005504	26	1.33e-03	10	
MF	antioxidant activity	GO:0016209	68	6.55e-07	20	
MF	poly(A) RNA binding	GO:0044822	1160	2.01e-04	105	
MF	identical protein binding	GO:0042802	1177	4.26e-11	127	100
MF	NAD binding	GO:0051287	48	1.79e-02	12	
MF	endopeptidase regulator activity	GO:0061135	170	9.65e-14	42	40
MF	phosphatidylcholine-sterol O-acyltransferase activator activity	GO:0060228	6	7.38e-03	5	
MF	alditol:NADP+ 1-oxidoreductase activity	GO:0004032	7	2.47e-02	5	
MF	structural constituent of cytoskeleton	GO:0005200	103	1.88e-06	23	24
MF	calcium ion binding	GO:0005509	689	8.46e-04		69
MF	actin-dependent ATPase activity	GO:0030898	12	4.79e-02	6	
MF	hydrolase activity	GO:0016787	2407	7.24e-16	229	208

source	term name<br>Biological pathways (KEGG)	term ID	n. of term genes	corrected p-value	KT	CT
keg	Other glycan degradation	KEGG:00511	18	4.68e-02		6
keg	Protein processing in endoplasmic reticulum	KEGG:04141	166	1.95e-04		27
keg	Phagosome	KEGG:04145	150	2.87e-02	23	
keg	Prion diseases	KEGG:05020	35	1.63e-03	11	
keg	Salmonella infection	KEGG:05132	84	5.78e-03	17	
keg	Lysosome	KEGG:04142	124	6.94e-06		25
keg	Regulation of actin cytoskeleton	KEGG:04810	219	2.86e-02	30	
keg	Fructose and mannose metabolism	KEGG:00051	33	3.26e-02	9	
keg	Amoebiasis	KEGG:05146	99	7.85e-03	18	17
keg	Staphylococcus aureus infection	KEGG:05150	52	7.42e-08	19	12
keg	Biosynthesis of amino acids	KEGG:01230	75	1.80e-02	15	
keg	Pathogenic Escherichia coli infection	KEGG:05130	55	3.41e-02	12	
keg	Carbon metabolism	KEGG:01200	113	9.80e-03	20	
keg	Proteasome	KEGG:03050	44	1.82e-06	16	
keg	Pertussis	KEGG:05133	75	4.07e-07	22	
keg	Glycolysis / Gluconeogenesis	KEGG:00010	67	1.57e-04	16	16
keg	Complement and coagulation cascades	KEGG:04610	79	2.35e-18	35	20

source	term name<br>Biological pathways (Reactome)	term ID	n. of term genes	corrected p-value	KT	CT
rea	The role of GTSE1 in G2/M progression after G2 checkpoint	REAC:8852276	61	1.33e-05	18	
rea	Vesicle-mediated transport	REAC:5653656	597	7.54e-05	70	67
rea	Activation of NF-kappaB in B cells	REAC:1169091	68	1.48e-05	19	
rea	PCP/CE pathway	REAC:4086400	94	1.71e-06	24	
rea	Metabolism of proteins	REAC:392499	1402	3.32e-03	130	117
rea	NIK-->noncanonical NF-kB signaling	REAC:5676590	60	1.00e-05	18	
rea	G2/M Checkpoints	REAC:69481	170	4.13e-02	25	
rea	Common Pathway of Fibrin Clot Formation	REAC:140875	22	1.85e-02	8	
rea	Hedgehog ligand biogenesis	REAC:5358346	66	1.98e-07	21	
rea	Asparagine N-linked glycosylation	REAC:446203	283	1.58e-03		37
rea	CDK-mediated phosphorylation and removal of Cdc6	REAC:69017	52	7.55e-07	18	
rea	Calnexin/calreticulin cycle	REAC:901042	26	3.38e-02		8
rea	EPH-Ephrin signaling	REAC:2682334	96	1.14e-03	20	
rea	Lipid digestion, mobilization, and transport	REAC:73923	108	5.41e-04	22	
rea	M Phase	REAC:68886	306	3.43e-02	38	
rea	Sema4D in semaphorin signaling	REAC:400685	28	1.89e-02	9	
rea	Detoxification of Reactive Oxygen Species	REAC:3299685	34	3.18e-04	12	9
rea	Response to elevated platelet cytosolic Ca2+	REAC:76005	137	4.11e-13	39	35
rea	Degradation of beta-catenin by the destruction complex	REAC:195253	84	5.47e-04	19	
rea	VEGFA-VEGFR2 Pathway	REAC:4420097	326	5.95e-03	42	
rea	Regulation of activated PAK-2p34 by proteasome mediated degradation	REAC:211733	51	5.25e-07	18	
rea	SCF(Skp2)-mediated degradation of p27/p21	REAC:187577	61	2.02e-06	19	
rea	Regulation of actin dynamics for phagocytic cup formation	REAC:2029482	100	2.87e-02	18	
rea	AUF1 (hnRNP D0) binds and destabilizes mRNA	REAC:450408	57	8.28e-09	21	12
rea	Antigen processing-Cross presentation	REAC:1236975	101	1.62e-06	25	
rea	Autodegradation of the E3 ubiquitin ligase COP1	REAC:349425	53	1.07e-06	18	
rea	Regulation of Complement cascade	REAC:977606	26	4.13e-08	14	
rea	Extracellular matrix organization	REAC:1474244	290	5.87e-09		49
rea	Signaling by Rho GTPases	REAC:194315	407	1.59e-03	51	
rea	Defective CFTR causes cystic fibrosis	REAC:5678895	62	2.74e-06	19	
rea	SCF-beta-TrCP mediated degradation of Emi1	REAC:174113	56	2.94e-06	18	
rea	ABC-family proteins mediated transport	REAC:382556	107	4.59e-04	22	
rea	Degradation of AXIN	REAC:4641257	56	2.94e-06	18	
rea	Regulation of RAS by GAPs	REAC:5658442	69	3.21e-06	20	
rea	Degradation of DVL	REAC:4641258	58	5.50e-06	18	
rea	Metabolism	REAC:1430728	2031	1.17e-08	200	160
rea	FCERI mediated NF-kB activation	REAC:2871837	115	5.74e-03	21	
rea	Termination of O-glycan biosynthesis	REAC:977068	25	2.47e-02		8
rea	Host Interactions of HIV factors	REAC:162909	133	2.30e-08	32	
rea	Downstream TCR signaling	REAC:202424	100	8.21e-03	19	
rea	Ubiquitin-dependent degradation of Cyclin D1	REAC:69229	52	9.04e-08	19	
rea	MAPK6/MAPK4 signaling	REAC:5687128	95	1.08e-05	23	
rea	Hh mutants that don't undergo autocatalytic processing are degraded by ERAD	REAC:5362768	57	7.04e-08	20	
